# Cytotoxic and Apoptosis-Inducing Activity of Triterpene Glycosides from *Holothuria scabra* and *Cucumaria frondosa* against HepG2 Cells

**DOI:** 10.3390/md12084274

**Published:** 2014-07-24

**Authors:** Juanjuan Wang, Hua Han, Xiangfeng Chen, Yanghua Yi, Hongxiang Sun

**Affiliations:** 1 Key Laboratory of Animal Virology of Ministry of Agriculture, College of Animal Sciences, Zhejiang University, Hangzhou 310058, China; E-Mails: wjuanjuanhaohao@126.com (J.W.); chenxiangfneg@163.com (X.C.); 2 School of Medicine, Tongji University, Shanghai 200092, China; E-Mail: hanhua@tongji.edu.cn; 3 Research Center for Marine Drugs, School of Pharmacy, Second Military Medical University, Shanghai 200433, China; E-Mail: yiyanghua@hotmail.com

**Keywords:** *Holothuria scabra*, *Cucumaria frondosa*, triterpene glycosides, echinoside A, cytotoxicity, structure-activity relationship, apoptosis, mechanism

## Abstract

The cytotoxic effects of thirteen triterpene glycosides from *Holothuria scabra* Jaeger and *Cucumaria frondosa* Gunnerus (Holothuroidea) against four human cell lines were detected and their cytotoxicity-structure relationships were established. The apoptosis-inducing activity of a more potent glycoside echinoside A (**1**) in HepG2 cells was further investigated by determining its effect on the morphology, mitochondrial transmembrane potential (Δψ*_m_*) and mRNA expression levels of the apoptosis-related genes. The results showed that the number of glycosyl residues in sugar chains and the side chain of aglycone could affect their cytotoxicity towards tumor cells and selective cytotoxicity. **1** significantly inhibited cell viability and induced apoptosis in HepG2 cells. **1** also markedly decreased the Δψ*_m_* and Bcl-2/Bax mRNA express ratio, and up-regulated the mRNA expression levels of Caspase-3, Caspase-8 and Caspase-9 in HepG2 cells. Therefore, **1** induced apoptosis in HepG2 cells through both intrinsic and extrinsic pathway. These findings could potentially promote the usage of these glycosides as leading compounds for developing new antitumor drugs.

## 1. Introduction

Conventional chemotherapeutics and targeted antineoplastic agents have been developed based on the simplistic notion that cancer constitutes a cell-autonomous genetic or epigenetic disease. In spite of their high antitumor efficacy, numerous currently used chemotherapeutic drugs exhibit considerable adverse side effects and cumulative toxicities including immunosuppressant, nervous and gastrointestinal injuries. Furthermore, the development of resistance to chemotherapy is considered a major hindrance to treatment of various cancers, as a notable proportion of tumors relapses and develops resistance, eventually resulting in multidrug resistance following exposure to multiple anticancer drugs with prevalent structure and mechanisms of action [1]. Apoptosis, a highly regulated programmed cell death, has become a matter of great interest in cancer therapy and oncology because of the high potential of various chemotherapeutic agents in inducing apoptosis in a variety of cancer cells [2]. Therefore, the discovery and identification of natural and synthetic products capable of inducing apoptosis in cancer cells have become an important goal of research in antitumor pharmacology and oncotherapy.

Marine-derived natural products contain a variety of chemotherapeutic compounds that have been shown to prevent the development of malignancies [3]. Several marine-derived sulfate triterpene glycosides exhibit dual antiangiogenic and antitumor effects [4,5], suggesting that those triterpene glycosides may be a rich source for cancer therapy agents. Sea cucumbers are soft-bodied worm-like echinoderms which belong to the class Holothuroidea [6]. They have economic importance in Asian countries, especially in China, where several species are used in traditional medicine or eaten as delicacies [7,8]. Sea cucumbers are gaining more attention due to their diverse structural features and bioactivities. Sea cucumbers’ triterpene glycosides (holothurins) were used as potential drugs in the pharmaceutical industry and as nutraceuticals in the food industry [9].

Thus far, more than 170 triterpene glycosides have been isolated from sea cucumbers [10]. The majority of sea cucumber glycosides are lanostane derivatives with an 18(20)-lactone aglycone and a carbohydrate chain linked to the C-3 of the aglycone [6]. The sugar chains of these glycosides have two to six monosaccharide residues including xylose, quinovose, glucose and 3*-O-*methylglucose, and sometimes 3-*O*-methylxylose, 3-*О*-methylquinovose, 3-*О*-methylglucuronic acid (MeGlc) and 6-*О*-acetylglucose. They may also contain one, two, or three sulfate groups [11,12]. Usually, triterpene aglycones exhibit 12, 16 and 17-hydroxy groups with a 9(11) or 7(8) double bond, of which the side chains diversify a lot by presenting double bond and oxidation to a certain extent [12,13]. All of these made up quite a lot of holothurin analogs.

The triterpene glycosides have been proved to be the main bioactive principles of sea cucumbers, with a wide spectrum of biological activities such as antifungal, cytotoxic, hemolytic, and immunomodulatory effects [14,15,16,17,18,19,20,21]. Sea cucumber triterpene glycosides have also been considered as responsible agents for the protection against some form of cancer. Therefore, many studies have been conducted to explore their anticancer effect. However, their structure-activity relationship and mechanisms for anticancer action have not been well elucidated [9]. Meanwhile, in finding therapeutics from natural products, always the preference is given to the compounds having high specificity toward the cancer cells, while minimizing the damage to normal cells. However, the selective cytotoxicity of sea cucumber glycosides in neoplastic *versus* normal cells have not yet been well studied. The special and systematic study on structure-cytotoxicity and selective cytotoxicity of sea cucumber triterpene glycosides using a series of purified and structurally consecutive analogs may be useful for further modification and optimization in developing new anticancer drugs.

**Figure 1:**
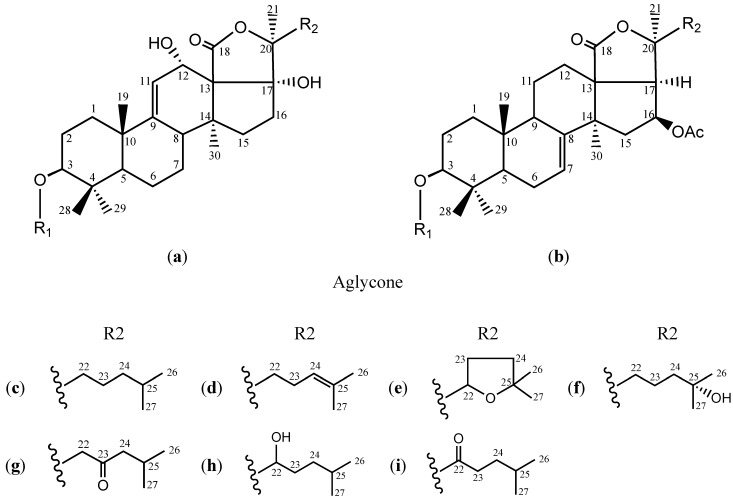
Chemical structures of triterpene glycosides **1**–**13** from *H. scabra* and *C. frondosa*. Glc: β-d-glucopyranosyl; MeGlc: 3-*O*-methyl-β-d-glucopyranosyl; Qui: β-d-quinovo-pyranosyl; Xyl: β-d-xylopyranosyl; S1: Qui-(1→2)-4-*O*-SO_3_Na-Xyl-; S2: MeGlc-(1→3)-Glc-(1→4)-Qui-(1→2)-4*-O-*SO_3_Na-Xyl-; S3: MeGlc-(1→3)-Xyl-(1→4)-Qui-(1→2)-4-*O*-SO_3_Na-Xyl-; S4: MeGlc-(1→3)-Xyl-(1→4)-[Xyl-(1→2)]-Qui-(1→2)-4-*O*-SO_3_Na-Xyl-; S5: MeGlc-(1→3)-Xyl-(1→4)-[Xyl-(1→2)]-Qui-(1→2)-4-*O*-SO_3_H-Xyl-; (**a**) and (**b**): Aglycone; (**c**–**i**): R2.

In this paper, thirteen structurally consecutive triterpene glycosides (Figure 1 and Table 1) isolated from *Holothuria scabra* Jaeger (**1**–**9**) and *Cucumaria frondosa* Gunnerus (**10**–**13**) were evaluated for their cytotoxic activities against neoplastic and normal cell lines to assess the contribution of the structural characteristics on the bioactivities and to consider the structural factors essential for the fundamental antitumor effects and selective cytotoxicities of these glycosides. Meanwhile, echinoside A (**1**), a more potent glycoside by comparing the effect on the growth of hepatoma carcinoma HepG2 cells and normal hepatocyte HL-7702 cells, was selected for further investigating its mechanism of apoptosis-inducing activity in HepG2 cells.

**Table 1:** Chemical structures of triterpene glycosides **1**–**13** from *H. scabra* and *C. frondosa*.

Glycosides	Aglycone	R1	R2	MF	MR	Reference
Echinoside A (**1**)	a	S2	c	C_54_H_87_O_26_NaS	1206	[22]
24-Dehydro echinoside A (**2**)		S2	d	C_54_H_85_O_26_NaS	1204	[23]
Holothurin B (**3**)		S1	e	C_41_H_62_O_17_NaS	881	[22]
Holothurin B4 (**4**)		S1	f	C_41_H_65_O_17_NaS	884	
HS-1 (**5**)		S2	g	C_54_H_85_O_27_NaS	1220	[24]
Holothurin A (**6**)		S2	e	C_54_H_85_O_27_NaS	1220	[22]
Holothurin A1 (**7**)		S2	h	C_54_H_87_O_27_NaS	1222	[24]
Scabraside D (**8**)		S2	f	C_54_H_87_O_27_NaS	1222	[23]
Scabraside B (**9**)		S2	i	C_54_H_85_O_27_NaS	1220	[25]
Frondoside A1 (**10**)	b	S3	c	C_55_H_87_O_25_NaS	1202	[26]
Frondoside A (**11**)		S4	c	C_60_H_95_O_29_NaS	1334	[26]
Frondoside A6 (**12**)		S5	c	C_60_H_95_O_29_HS	1312	[26]
24-Dehydro frondoside A6 (**13**)		S5	d	C_60_H_93_O_29_HS	1310	

The chemical structures of a–i and S1–S5 see Figure 1.

## 2. Results and Discussion

### 2.1. Cytotoxicity against Tumor Cells and Structure-Activity Relationships

The cytotoxic activity of triterpene glycosides **1**–**13** towards human hepatoma (HepG2), human cervical cancer (HeLa) and human leukemia (K562) cells were measured using MTT assay. As shown in Table 2, all thirteen glycosides exhibited high cytotoxic activities against three human tumor cell lines with the IС_50_ values being 1−10 μg/mL. However, there were still differences in cytotoxic activity for different compounds with special chemical structures, especially against K562 cells.

**Table 2:** Cytotoxicity of triterpene glycosides **1**–**13** against four human cell lines *in vitro*.

Glycosides	IC_50_ (μg/mL)				Ratio (HL-7702/HepG2)
	HeLa	K562	HepG2	HL-7702	
Echinoside A (**1**)	1.25 ± 0.16	1.61 ± 0.11	1.50 ± 0.08	3.12 ± 0.65 *	2.08
24-Dehydro echinoside A (**2**)	2.06 ± 0.27	6.15 ± 0.24	1.95 ± 0.09	3.75 ± 0.40 **	1.92
Holothurin B (**3**)	2.05 ± 0.11	3.64 ± 0.04	1.79 ± 0.02	2.69 ± 0.45 *	1.50
Holothurin B4 (**4**)	2.71 ± 0.54	3.55 ± 0.53	2.71 ± 0.12	4.58 ± 0.54 **	1.69
HS-1 (**5**)	3.20 ± 0.32	12.95 ± 1.54	6.10 ± 0.33	7.19 ± 0.20 **	1.18
Holothurin A (**6**)	3.76 ± 0.47	8.94 ± 0.01	3.46 ± 0.33	3.85 ± 0.32	1.11
Holothurin A1 (**7**)	2.84 ± 0.09	6.50 ± 0.01	2.90 ± 0.21	3.59 ± 0.05 **	1.24
Scabraside D (**8**)	3.84 ± 0.42	10.06 ± 1.06	3.33 ± 0.33	4.40 ± 0.22 **	1.32
Scabraside B (**9**)	4.44 ± 0.85	11.85 ± 2.26	7.29 ± 0.62	11.03 ± 1.37 *	1.51
Frondoside A1 (**10**)	2.03 ± 0.17	2.21 ± 0.56	1.91 ± 0.03	4.96 ± 1.10 **	2.60
Frondoside A (**11**)	3.30 ± 0.05	3.76 ± 0.05	4.14 ± 0.57	5.42 ± 0.98	1.31
Frondoside A6 (**12**)	3.75 ± 0.51	6.59 ± 0.11	2.53 ± 0.66	6.05 ± 0.67 **	2.39
24-Dehydro frondoside A6 (**13**)	3.16 ± 0.34	5.61 ± 1.05	3.57 ± 0.54	7.55 ± 0.83 **	2.12
CDDP (positive control)	3.41 ± 0.37	2.99 ± 0.24	2.05 ± 0.13	4.48 ± 0.41 ***	2.18

The values are presented as means ± SD (*n* = 3). Significant differences with the IC_50_ values against HepG2 cell were designated as * *p* < 0.05, ** *p* < 0.01, and *** *p* < 0.001.

In order to evaluate the contribution of the sugar moieties to the cytotoxicity toward tumor cells, we compared the cytotoxicity of three pairs of holothurins including **3** and **6**, **4** and **8**, **10** and **11**, respectively. Two compounds per pair of glycosides possess the same aglycone with only a slight difference in the sugar chain attached to C-3 of aglycone, *i.e.*, the latter differs from the former by the addition of one or two monosaccharide residues (Figure 1). All six glycosides strongly inhibited the growth of HepG2, HeLa and K562 cells. Of two glycosides per pair, the former had more potent cytotoxic activity than the latter, suggesting that the cytotoxic potential of these glycosides toward tumor cells could decrease with the increased number of monosaccharide residues at the sugar moieties. Yan *et al.* [22] reported the cytotoxicity of five triterpene glycosides **1**, **2**, **3**, **6** and echinoside B against human gastric cancer MKN-45 cells with their IC_50_ values being 1.86, 1.60, 1.59, 2.37 and 0.18 μmol/L, respectively. Compound **6** differs from **3**, and **1** from echinoside B by the addition of two monosaccharides. The cytotoxicities of **6** and **1** against MKN-45 cells were lower than those of **3** and echinoside B, respectively. It also was reported that **3** had more potent cytotoxic activity than **6** against human leukemia HL-60 and human hepatoma BEL-7402 cells [22].

Zou *et al.* [27,28] determined cytotoxicity of several triterpene glycosides from *Mensamaria intercedens* against ten human tumor cell lines and found that intercedenside H exhibited more significant antitumor activity than intercedenside C, especially on human breast cancer MCF-7, ovarian cancer IA9s, renal cancer CAKI-1, and melanoma SK-MEL-2 cells. Intercedenside C differs from intercedenside H by only the addition of one β-d-xylopyranosyl group. Such a correlation between activity and chemical structure is consistent with our findings.

The holothuroid triterpene glycosides have strong membranolytic action against cellular and model membranes, which is the basis of their hemolytic, antifungal and cytotoxic activities [19]. The chemical structures including lanostane aglycone moiety, the type and number of glycosyl groups in sugar chain units, and some special functional groups influenced the membranolytic action of the holothuroid triterpene glycosides against cellular and model membranes [19]. Therefore, the information about relationships between hemolytic activity and the sugar chain might be useful for assessing the potential contribution of the sugar chains to the cytotoxic activity. Kalinin *et al.* [29] demonstrated that the presence of 3-*O*-methyl at the terminal monosaccharide unit greatly increased the hemolysis. Avilov *et al.* [30] explained the reason from the respect of evolution. It was confirmed by the fact that cucumarioside A2-2 possessed more active antitumor activity than cucumarioside A4-2 [31], with the only structural difference being the presence (A2-2) or absence (A4-2) of 3-*O*-methyl group at the terminal monosaccharide unit. The sulfation of the sugar chain is also a significant factor related to bioactivity. The presence of a sulfate group at C-4 of the first xylose residue attached to C-3 of aglycone increased the effect against membranes and hemolytic activity [29], which was also confirmed by findings that the cytotoxicity of okhotosides B2 (IC_50_ 13.0 μg/mL) against HeLa cells were more potent than that of okhotosides B3 (IC_50_ 17.8 μg/mL) [32]. The presence of a sulfate at C-4 of the first xylose in branched pentanosides having 3-*O*-methyl group at the terminal monosaccharide could increase activity. However, the same sulfate can decrease the activity of branched pentanosides, which have glucose as the terminal residue [33]. Recently, Zhao *et al.* [34] compared the antitumor activities of echinoside A and *ds*-echinoside A *in vitro* and *in vivo*. *Ds*-echinoside A is a non-sulfated triterpene glycoside, which was derived from the desulfurization reaction of echinoside A. *Ds*-echinoside A more strongly inhibited the viability of HepG2 cells, induced cell cycle arrest and apoptosis *in vitro*, and inhibited tumor growth *in vivo* than **1** [34]. These results suggested that the number of sugar residues, the presence of the 3-*O*-methyl group at the terminal monosaccharide units and sulfate group in the sugar side chains could affect the cytotoxic activities of sea cucumber triterpene glycosides.

Sea cucumber triterpene glycosides have quite complicated structures and may be distinguished by many relatively independent characters, such as the position of double bond in the cyclic system of the aglycone, the number and position of double bonds in the side chain of aglycone, as well as the number and different position of hydroxyl, epoxy group, acetyl and oxo groups in aglycone, except for the sugar moieties. The effect of the side chain in aglycone of glycosides on the cytotoxicity toward tumor cells also discussed. Among 13 glycosides, **5** and **9** are only the two compounds containing carbonyl group at the side chain of aglycone. The IC_50_ values of **5** and **9** against HeLa, HepG2, and K562 cells were significantly higher than those of other 11 triterpene glycosides. The chemical structures of **5**, **9** and **1** are very similar with the exception that an additional carbonyl group was present in the former two compounds. Further comparison of their cytotoxicity against HeLa, HepG2 and K562 cells showed that **5** and **9** was notably weaker than **1**, suggesting that the presence of a carbonyl group at the side chain of the aglycone could significantly decrease their cytotoxicities.

The same common structural features are exhibited by **1** and **6**, except for the addition of epoxy group at the side chains of aglycone for **6**. The IC_50_ values of **6** against HeLa, HepG2 and K562 cells were higher than those of **1** by 2−5 folds. Differing from **1** are **7** and **8**, due to the addition of one hydroxyl group. Among these three glycosides, the cytotoxic activities of **1** toward HeLa, HepG2 and K562 cells were also stronger than that of **7** and **8** by 2–5 times. These results suggested that the presence of hydroxyl and epoxy groups at the side chains of aglycone could decrease the cytotoxic activity towards tumor cells. It was also confirmed by the results obtained in this study that the cytotoxic activities of **2** toward HeLa, HepG2 and K562 cells were significantly stronger than that of **8**. Interestingly, **2** and **8** are glycosides of the holostane type with identical carbohydrate chains; **2** has 24(25)-double bond, while **8** has a hydroxyl group at C-25 in a side chain. However, **2** and **7** differed from each other in structure of their side chains: **2** has a 24(25)-double bond and **7** has a hydroxyl group at C-21. Zhao *et al.* [35] reported the cytotoxic effects of **2** and **7** on HepG2, B16, CaCo-2, HeLa, P388 and S180 cells and the anti-metastatic activity *in vitro* and *in vivo*, and found that **2** had more potent cytotoxic and anti-metastatic activity than **7**. Above all, the carbonyl, hydroxyl and epoxy groups at the side chain of the aglycone played a negative role in inhibiting tumors to some extent.

### 2.2. Selective Cytotoxicity on Tumor and Normal Cells

To compare the differential cytotoxicities of **1**–**13** in neoplastic *versus* proliferating normal cells, their cytotoxic activities on HL-7702 cells were also determined using the MTT assay, and the results are shown in Table 2. All thirteen glycosides also showed a marked cytotoxic activity against HL-7702. We calculated the ratios of the IC_50_ values against HL-7702 and HepG2 cells to evaluate their selective cytotoxicity on tumor cell lines. Interestingly, we found that some more active compounds against tumor cell lines such as **1** and **10** had higher ratios, even more than **2**, which indicated that these two glycosides had more potential to be explored as novel antitumor drugs with lower side effect. In contrast, the triterpene compounds with lower cytotoxicities against tumor cells seemed to be more sensitive to normal cells. The trend of cytotoxicity of compounds **1**–**13** towards human normal renal NRK cells was consistent with that against HL-7702 cells (data not shown). Furthermore, we also found that the selective cytotoxicity of these glycosides against tumor cell lines was closely related to their chemical structures.

As shown in Table 2, four frondosides, with the exception of **11**, exhibited the better selective cytotoxicity towards tumor cells compared to other nine glycosides. Evidently, **11** differs from **10** by an additional xylose. Although there was no significant difference in cytotoxic activities against HL-7702 cells between **11** and **10**, the cytotoxicity of **10** on three tumor cells was more significant than that of **11**. Therefore, the cytotoxicity of **10** was more selective than that of **11** in neoplastic *vs.* normal cells. This was consistent with the aforementioned results that the cytotoxic potential of these glycosides toward tumor cells could decrease with the increased number of monosaccharide of the sugar moieties. In addition, **1** and **2** also possessed the better selective cytotoxicity on tumor cell lines compared to the other seven glycosides with the same aglycone. Compounds **1** and **2** lack the oxygen-containing groups, while the other seven glycosides contain the carbonyl, hydroxyl or epoxy groups at the side chain of the aglycone. These results suggested that the presence of these oxygen-containing groups also had an influence on the selective cytotoxicity of these glycosides towards tumor cell lines.

### 2.3. Apoptosis-Inducing Effect of 1 on HepG2 Cells

Compound **1** possessed the strongest antitumor activity and the lower cytotoxic effect on normal cells among thirteen glycosides. It has been shown that **1** is a new non-intercalative Top2 inhibitor targeting Top2a by unique interference in DNA binding and impairment of Top2-mediated DNA cleavage and relegation [36]. Compound **1** has also been proved to exhibit marked anticancer activity in HepG2 cells by blocking cell-cycle progression and inducing apoptosis through the intrinsic (mitochondrial) pathway [34]. However, the contribution of the extrinsic (death receptors) pathway in the apoptosis-inducing effect of **1** has not yet been made clear. Therefore, we further investigated the growth-inhibiting and apoptosis-inducing activity of **1** and its mechanisms using HepG2 cells.

**Figure 2:**
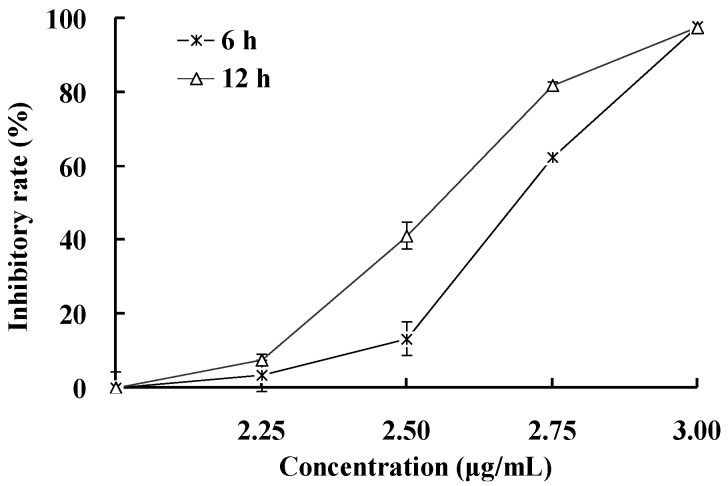
Effect of **1** on cell viability of HepG2 cells. HepG2 cells were treated with the various concentrations of **1** for 6 and 12 h, and cell viability was measured using the MTT assay. The values are presented as means ± SD (*n* = 5).

To determine the effects of **1** on the viability of HepG2 cell, the cells were cultured with **1** at different concentrations for 6 h and 12 h, and cell viability was measured using MTT assay. As shown in Figure 2, **1** significantly inhibited the survival of HepG2 cells in a time- and concentration-dependent manner within narrow effective concentration range 2.25−3 μg/mL. Compound **1** hardly influenced cell viability at the concentration of 2.25 μg/mL. However, HepG2 cells were completely inhibited by **1** at a concentration of 3 μg/mL. These results suggested that HepG2 cell was quite sensitive to **1**.

**Figure 3:**
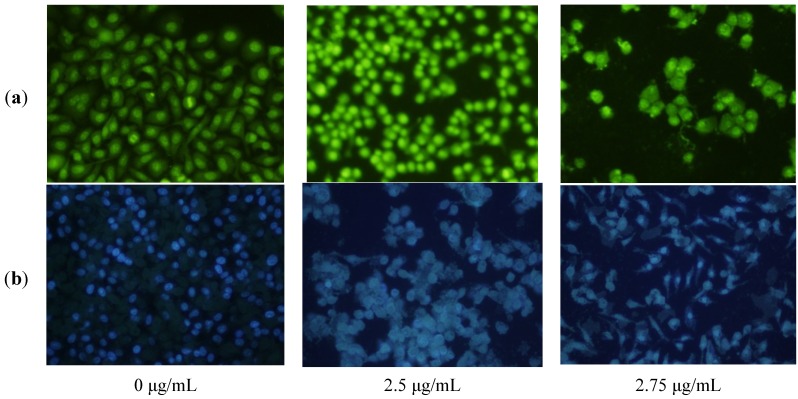
Morphological changes of HepG2 cells after treatment with **1** for 8 h. (**a**) Morphological changes visualized under a fluorescence microscope after acridine orange (OA) staining; (**b**) Morphological changes visualized under a fluorescence microscope after Hoechst 33342 staining. The figures shown are representative of three independent experiments.

The induction of apoptosis in cancer cells has emerged as an exciting possibility for the development of selective cancer therapies [37]. Apoptosis is characterized by distinct morphological features such as cell shrinkage, loss of contact with neighboring cells, formation of cytoplastic vacuoles, chromatin condensation, nuclear-membrane blebbing, oligonucleosomal DNA fragmentation, and finally breakdown of the cell into smaller units (apoptotic bodies). To further characterize the anticancer activity of **1**, disorganization of the nucleus with chromatin changes in HepG2 cells treated with **1** at the concentration of 2.5 and 2.75 μg/mL for 8 h was characterized using acridine orange (OA) and Hoechst 33342 staining under fluorescence microscope. As shown in Figure 3a, after staining with AO, the DNA in the nucleus of control cells had homogeneously *Kelly* fluorescence, while **1**-treated cells showed typical apoptosis features characterized by volume reduction, chromatin condensation, and nuclear fragmentation with densely *Kelly* fluorescence stain, and appearance of apoptotic bodies. The Hoechst 33342 staining assay showed that cells also demonstrated apoptotic features such as nuclear shrinkage, chromatin condensation, and fragmentation in a concentration-dependent manner after treatment with **1** for 8 h (Figure 3b). These results indicated that **1** could induce apoptosis in HepG2 cells.

**Figure 4:**
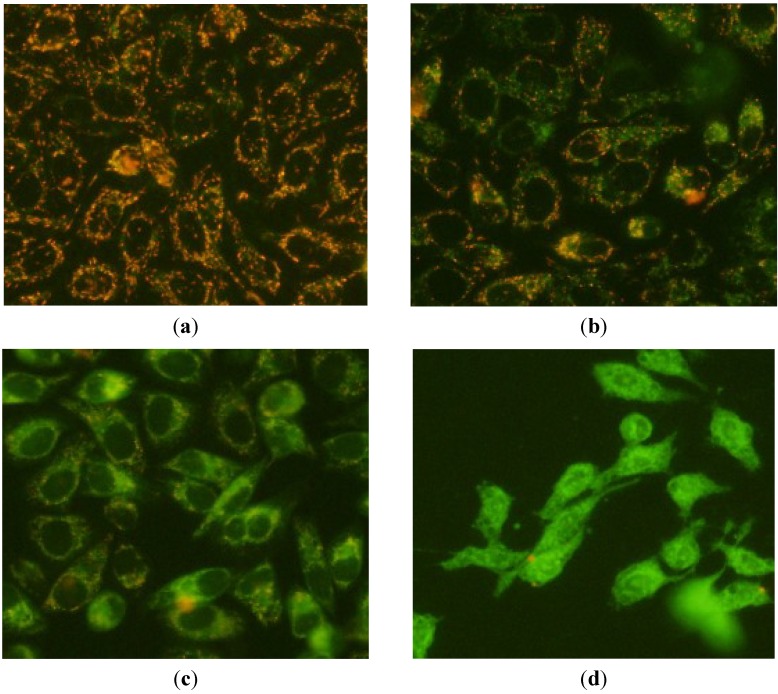
Mitochondrial transmembrane potential changes of **1**-treated HepG2 cells stained with JC-1 at the concentrations of 0 μg/mL (**a**); 2.5 μg/mL (**b**); 2.75 μg/mL (**c**) and 3.0 μg/mL (**d**). The figures shown are representative of three independent experiments.

The mitochondrial transmembrane potential (Δψ*_m_*) collapse has been shown to play an essential role in mediating apoptosis. JC-1 is an ideal fluorescent probe to detect the change of Δψ*_m_*, which exhibits red fluorescence as JC-1 aggregates in mitochondrial matrix under high mitochondrial potential but green fluorescence as monomeric form under low potential. Therefore, we measured the Δψ*_m_* in **1**-treated HepG2 cells and control cells using JC-1 staining, and the results were shown in Figure 4. The aggregated JC-1 within normal mitochondria in HepG2 cells was dispersed to the monomeric form (green fluorescence) after treatment with **1** for 6 h. Moreover, the intension of green fluorescence in HepG2 cells significantly enhanced with the increased concentration of **1**. After treatment with **1** at 3 μg/mL, the red fluorescence disappeared and pure green fluorescence only occurred in HepG2 cells. These results suggest that **1** significantly decreased the Δψ*_m_* in HepG2 cells and further confirmed the participation of a mitochondria-related mechanism in the apoptosis in **1**-treated HepG2 cells.

**Figure 5:**
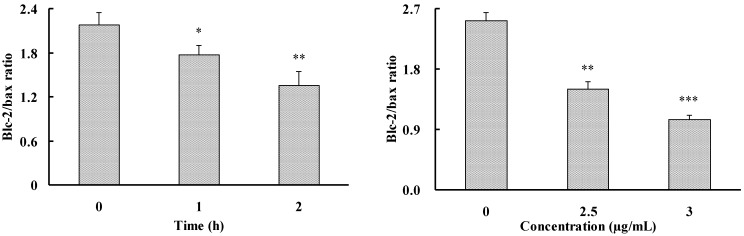
Effect of **1** on the ratio of expression level between Bcl-2 and Bax mRNA in HepG2 cells. HepG2 cells were incubated with **1** at the various concentrations for the different time. The mRNA expression levels of β-actin, Bcl-2, and Bax were detected by RT-PCR using specific primers. The values are presented as means ± SD (*n* = 3). Significant differences with the untreated control group were designated as * *p* < 0.05, ** *p* < 0.01 and *** *p* < 0.001.

The Bcl-2 family members can alter the permeability of mitochondrial membrane, and release cytochrome c or activate caspase cascade. There are two classes of regulatory proteins in the Bcl-2 family that have opposite effects on apoptosis: The anti-apoptotic members (Bcl-2 and Bcl-XL) protect cells against some forms of apoptosis, whereas the pro-apoptotic members (Bax and Bcl-xS) promote programmed cell death. As decrease of the ratio between anti-apoptotic gene Bcl-2 and pro-apoptotic gene Bax mRNA expression is widely considered as a hallmark of apoptosis, the expression level of Bcl-2 and Bax mRNA in HepG2 cells were first detected. Compound **1** significantly decreased the ratio of Bcl-2/Bax mRNA expression in HepG2 cells in a time- and concentration-dependent manner (Figure 5), suggesting that **1** may induce apoptotic cell death by altering the gene expression level of these two Bcl-2 family members.

**Figure 6:**
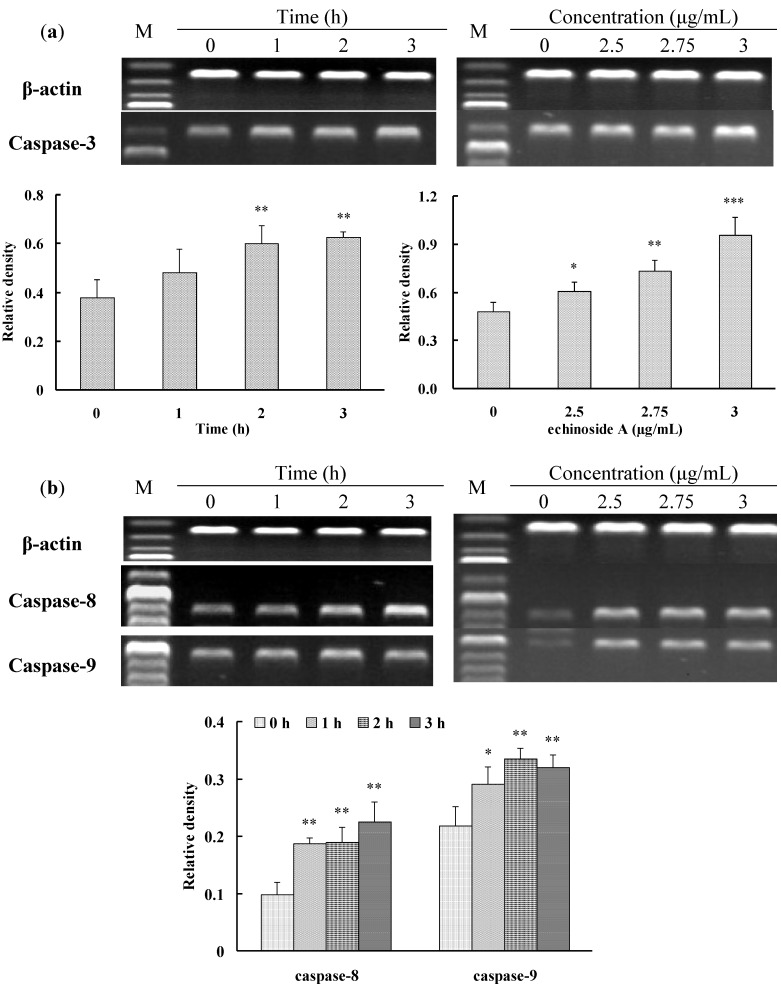
Effects of **1** on the mRNA expression level of Caspase-3, Caspase-8 and Caspase-9 in HepG2 cells. HepG2 cells were incubated with **1** at the various concentrations for the different time. The mRNA expression levels of β-actin, Caspase-3, Caspase-8 and Caspase-9 were detected by RT-PCR using specific primers. (**a**) The mRNA expression of Caspase-3 in HepG2 cells; (**b**) The mRNA expression of Caspase-8 and Caspase-9 in HepG2 cells. The values are presented as means ± SD (*n* = 3). Significant differences with the untreated control group were designated as * *p* < 0.05, ** *p* < 0.01 and *** *p* < 0.001.

Caspases play an important role in cell apoptosis. Activation of caspase appears to be directly responsible for many molecular and structural changes in apoptosis. The caspase-dependent process is associated with two pathways of anticancer drug-induced apoptosis, the extrinsic (death receptor) pathway and the intrinsic (mitochondrial) pathway [38]. The extrinsic pathway is initiated by death ligands, such as FasL, leading to cleavage of pro-Caspase-8 to its active form, which subsequently activates downstream effectors such as Caspase-3. The intrinsic pathway is characterized by mitochondria dysfunction, resulting in the reduction of mitochondrial transmembrane potentials, mitochondrial translocation of the pro-apoptotic Bax protein, cytochrome c release and activation of downstream effector caspases such as Caspase-9 [39,40]. Caspase-3 is one of the downstream effectors of the caspase family, and is considered to involve both the mitochondrial apoptotic pathway and the death receptor pathway. To demonstrate the role of these two pathways in apoptosis of HepG2 cells induced by **1**, we further measured the expression levels of Caspase-3, Caspase-8 and Caspase-9 mRNA. As shown in Figure 6a, compared to control cells, the expression level of Caspase-3 mRNA in HepG2 cells was markedly up-regulated by **1** in time- and dose-dependent manners. Compound **1** also significantly up-regulated the expression levels of Caspase-8 and Caspase-9 mRNA in HepG2 cells. However, there were no significant differences in the expression level of Caspase-8 and Caspase-9 mRNA among HepG2 cells treated with **1** at different concentrations for 2 h (Figure 6b), which were similar to the observed rapid effects among 2.5–3 μg/mL in the MTT assay. These results suggested that **1** induced the apoptotic cell death in HepG2 cells through the activation of extrinsic and intrinsic apoptosis pathways.

## 3. Experimental Section

### 3.1. General Details

3-(4,5-Dimethylthiazol-2-yl)-2,5-diphenyltetrazolium bromide (MTT), acridine orange (AO), *cis*-diamminedichloroplatinum (CDDP, positive drug) and Hoechst 33342 were purchased from Sigma Chemical Co., Saint Louis, MO, USA; RPMI-1640 medium and fetal calf serum (FCS) were from Gibco, Grand Island, NY, USA; JC-1 mitochondrial potential sensors (JC-1) and Trizol reagent was obtained from Invitrogen Co., Carlsbad, CA, USA; revertAid™ M-MuLV reverse transcriptase, diethylpyrocarbonate (DEPC), ribonuclease inhibitor, Oligo(dT)18 and standard DNA marker were from Sangon, Shanghai, China.

Thirteen pure triterpene glycosides (**1**–**13**, Figure 1) were isolated from *H. scabra* Jaeger (**1**–**9**) and *Cucumaria frondosa* Gunnerus (**10**–**13**) (Holothuriidae) as previously described [22,23,24,25,26]. Each of the isolates was subjected to detailed spectroscopic analysis (IR, EI-MS, ESI-MS, HRESI-MS, ^1^H-NMR, ^13^C-NMR, ^1^H-^1^H COSY, DQCOSY, TOCSY, HMQC, HMBC and NOESY) to identify their chemical structures. The purity of each triterpene glycoside was determined to be >98% using a Symmetry^®^ C18 column (250 mm × 4.6 mm i.d., particle size 5 μm) and Waters 2996 PDA detector on the Water 600E HPLC instrument. A stock compound solution with a concentration of 1 mg/mL was prepared by dissolving in phosphate buffered solution (PBS). The solution was sterilized by passing it through a 0.22 μm Millipore filter, and then diluted with RPMI-1640 medium to the desired concentrations before use.

### 3.2. Cell Lines

Human hepatoma (HepG2), human cervical cancer (HeLa), human leukemia (K562) and human liver (HL-7702) cells were obtained by Shanghai Institute of Biochemistry and Cell Biology, and maintained in the logarithmic phase of growth in RPMI-1640 complete medium supplemented with 2 mM l-glutamine (Sigma Chemical Co., Saint Louis, MO, USA), 100 IU/mL penicillin, 100 μg/mL streptomycin and 10% FCS at 37 °C under humidified air with 5% CO_2_.

### 3.3. Cell Viability Assay

The effect of compounds **1**–**13** on the viability of HepG2, HeLa, K562 and HL-7702 cells was determined by MIT assay as described previously [41]. Briefly, the tumor and normal cells were seeded at 1 × 10^4^ cells/well in a 96-well microtiter plate and incubated at 37 °C in a humidified atmosphere with 5% CO_2_. After 24 h, compounds **1**–**13**, the positive drug CDDP or RPMI-1640 medium were added into each well, and the plates were incubated at 37 °C for the indicated time. Each concentration was repeated four wells. 50 μL of MTT solution (2 mg/mL) was added to each well 4 h before incubation end, and incubated further for 4 h. The plates were centrifuged (1400× *g*, 5 min) and the untransformed MTT was removed carefully by pipetting. To each well, 150 μL of DMSO was added and the absorbance was evaluated in an ELISA reader at 570 nm after 15 min. The inhibitory rates and 50% inhibitory concentrations (IC_50_) values towards cell viability were calculated by NDST software [42]. Each test was performed in triplicate.

### 3.4. Fluorescence Microscope Observation

HepG2 cells were seeded at 1 × 10^5^ cells/mL into 24-well plates and then incubated at 37 °C in a humidified atmosphere with 5% CO_2_. After 24 h, the cells were treated with **1** at the final concentrations of 0, 2.5 and 2.75 μg/mL for 8 h. After washed twice with PBS, cells were stained with 100 μL acridine orange (AO) solution (10 μg/mL) or Hoechst 33342 solution (5 μg/mL) for 30 min, and then visualized by fluorescence microscope (Olympus, Tokyo, Japan) with 488 nm stimulation and 500–520 nm emission or 350 nm stimulation and 460 nm emission, respectively.

### 3.5. Measurement of the Mitochondrial Transmembrane Potentials (Δψ_m_)

After treatment with **1** at the concentration of 0, 2.5, 2.75 and 3 μg/mL for 6 h, the cells were harvested, washed twice with PBS, and then incubated with 500 μL of JC-1 staining solution (5 μg/mL) at 37 °C for 30 min [43]. The stained cells were rinsed twice with PBS and resuspended in medium. The Δψ*_m_* was monitored by determining the relative amounts of dual emissions from mitochondrial JC-1 monomers (green fluorescence) to aggregates (red fluorescence) using an Olympus fluorescent microscope under Argon-ion 488 nm laser excitation.

### 3.6. RT-PCR Analysis

Total RNA was extracted with TRIzol reagent (Invitrogen, Grand Island, NY, USA) according to the manufacturer’s instructions, and reverse transcription was performed as previously [44]. PCR was performed for multiple cycles using a PTC-200 thermal cycler (Bio-Rad Laboratories, Inc., Berkeley, California, USA) with the following program of denaturation at 94 °C for 1 min, annealing for 50 s, and elongation at 72 °C for 0.5 min. The specific primers, amplified cycles and annealing temperature of each gene were listed in Table 3. Semi-quantitative RT-PCR was performed using β-actin as an internal control to normalize gene expression for the PCR templates. The PCR products were analyzed by electrophoresis on a 1.5% agarose gel containing goldview (5 μL/100 mL), and the amplified bands were visualized and photographed using JS-680B Gel Documentation and Analysis System (Shanghai Peiqing Science and Technology Co., Ltd., Shanghai, China). The size of the amplified fragments was determined by comparison with a standard DNA marker.

**Table 3:** Primer sequences and amplification for expected PCR products.

Gene	Primer Sequence	Product Size (bp)	Annealing Temperature (°C)	Cycle
β-Actin	5′-CTGTCTGGCGGCACCACCAT-3′ 5′-GCAACTAAGTCATAGTCCGC-3′	254	54	19
Bcl-2	5′-AGATGTCCAGCCAGCTGCACCTGAC-3′ 5′-AGATAGGCACCCAGGGTGATGCAAGCT-3′	367	57	33
Bax	5′-AAGCTGAGCGAGTGTCTCAAGCGC-3′ 5′-TCCCGCCACAAAGATGGTCACG-3′	366	66	26
Caspase-3	5′-TTTGTTTGTGTGCTTCTGAGCC-3′ 5′-ATTCTGTTGCCACCTTTCGG-3′	400	54	27
Caspase-8	5′-GGGACAGGAATGGAACACACTTGG-3′ 5′-TCAGGATGGTGAGAATATCATCGCC-3′	558	66	26
Caspase-9	5′-AACAGGCAAGCAGCAAAGTT-3′ 5′-TCCATCTGTGCCGTAGACAG-3′	511	54	25

### 3.7. Statistical Analysis

Data were expressed as mean ± SD and examined for their statistical significance of difference with the Student’s *t*-test. *p*-values of less than 0.05 were considered to be statistically significant.

## 4. Conclusions

In the present study, we report the structure-cytotoxic activity relationships for the sea cucumber triterpene glycosides using thirteen purified and structurally consecutive analogs from *H. scabra* and *C. frondosa*. The number of glycosyl residues in the sugar chains and the side chain in aglycone could affect not only the cytotoxicity towards tumor cells, but also the selective cytotoxicity in neoplastic *versus* normal cells of these glycosides. The information about this structure-function relationship might be useful for further modification and optimization in developing new anticancer drugs. Meanwhile, our study demonstrates that **1** induced apoptosis in HepG2 cells through both the intrinsic and extrinsic pathway and could act as a novel antitumor drug with better selective cytotoxicity.
